# Prediction of spatial distribution characteristics of ecosystem functions based on a minimum data set of functional traits of desert plants

**DOI:** 10.3389/fpls.2023.1131778

**Published:** 2023-06-02

**Authors:** Yudong Chen, Jinlong Wang, Lamei Jiang, Hanpeng Li, Hengfang Wang, Guanghui Lv, Xiaotong Li

**Affiliations:** ^1^ College of Ecology and Environment, Xinjiang University, Urumqi, China; ^2^ Key Laboratory of Oasis Ecology of Education Ministry, Xinjiang University, Urumqi, China; ^3^ Xinjiang Jinghe Observation and Research Station of Temperate Desert Ecosystem, Ministry of Education, Jinghe, China

**Keywords:** arid regions, random forest, regression kriging, semi-variable functions, spatial variation

## Abstract

The relationship between plant functional traits and ecosystem function is a hot topic in current ecological research, and community-level traits based on individual plant functional traits play important roles in ecosystem function. In temperate desert ecosystems, which functional trait to use to predict ecosystem function is an important scientific question. In this study, the minimum data sets of functional traits of woody (wMDS) and herbaceous (hMDS) plants were constructed and used to predict the spatial distribution of C, N, and P cycling in ecosystems. The results showed that the wMDS included plant height, specific leaf area, leaf dry weight, leaf water content, diameter at breast height (DBH), leaf width, and leaf thickness, and the hMDS included plant height, specific leaf area, leaf fresh weight, leaf length, and leaf width. The linear regression results based on the cross-validations (*FTEI_W - L_*, *FTEI_A - L_*, *FTEI_W - NL_*, and *FTEI_A - NL_*) for the MDS and TDS (total data set) showed that the *R^2^* (coefficients of determination) for wMDS were 0.29, 0.34, 0.75, and 0.57, respectively, and those for hMDS were 0.82, 0.75, 0.76, and 0.68, respectively, proving that the MDSs can replace the TDS in predicting ecosystem function. Then, the MDSs were used to predict the C, N, and P cycling in the ecosystem. The results showed that non-linear models RF and BPNN were able to predict the spatial distributions of C, N and P cycling, and the distributions showed inconsistent patterns between different life forms under moisture restrictions. The C, N, and P cycling showed strong spatial autocorrelation and were mainly influenced by structural factors. Based on the non-linear models, the MDSs can be used to accurately predict the C, N, and P cycling, and the predicted values of woody plant functional traits visualized by regression kriging were closer to the kriging results based on raw values. This study provides a new perspective for exploring the relationship between biodiversity and ecosystem function.

## 1 Introduction

Plant functional traits are morphological, physiological, and life history traits that indirectly affect plant fitness (Violle et al., 2007). In natural ecosystems, plants adapt to external changes by the changes of traits such as height, leaf area, leaf mass, leaf longevity, seed size, and seed dispersal mode, which may also lead to changes in ecosystem functions (Li et al., 2008; Messier et al., 2010; Albert et al., 2012; Kraft et al., 2015). However, plant species are diverse in nature, and plant functional traits are influenced by factors such as climate change and human disturbance (He et al., 2018). Therefore, using plant functional traits to reflect and predict changes in plant community and ecosystem function is of great importance.

Many studies have explored the relationship between plant functional traits and ecosystem processes or functions. Most researchers believe that the relative biomass of dominant species in plant communities and their specific traits dominate the dynamics of ecosystem processes in time and space (Vile et al., 2006; Catorci et al., 2014; Cavanaugh et al., 2014; Lohbeck et al., 2015). For example, Grime (1998) proposed the “mass ratio hypothesis”, arguing that plant functional traits can be used to predict ecosystem functions or processes (Diaz et al., 2007; Niu et al., 2010; Diaz et al., 2016). Furthermore, some scholars held that the change of one plant functional trait may lead to changes in multiple ecosystem functions, and one ecosystem function may be simultaneously affected by multiple plant functional traits (Temmerman et al., 2005; Kearney and Fagherazzi, 2016; Sun et al., 2020).

At present, there are two main ways to study the functional traits of plant communities. One is to use community functional parameters based on plant functional traits, for example, the community weighted mean (CWM) of plant functional traits, which is calculated using the weighted average of functional traits and relative abundances of species (Kattge et al., 2011; Zhang et al., 2011b; Zhang et al., 2020a). The other is to use plant functional trait diversity, for example, the size, range, and distribution of plant functional trait values in a community, which is considered important for biodiversity (Finegan et al., 2015; Huang et al., 2019b; Lin et al., 2022). Studies have shown that community functional parameters based on plant functional traits and plant functional trait diversity can influence plant community structure and ecosystem functions or processes (Flynn et al., 2011; Mei et al., 2017). However, the parameters are numerous, redundant, and cumbersome. Therefore, the selection of representative parameters that play an important role in ecosystem functioning has become the key to current research.

With the in-depth study of ecosystem functions, researchers gradually realize that ecosystems provide multiple ecosystem functions simultaneously, i.e. ecosystem multifunctionality (Pasari et al., 2013; Garland et al., 2020). Most previous studies have focused on the effects of a single trait on the functions of a single ecosystem (Fortunel et al., 2009; Zuo et al., 2016; Pakeman and Fielding, 2020) and the quantification of plant functional trait diversity and ecosystem functions (Petchey and Gaston, 2002; Laliberte and Legendre, 2010; Isbell et al., 2011). In recent years, quantitative analysis of the relationship between multiple plant functional traits and multiple functions of single ecosystems has been sought after (Wei et al., 2016a; Wei et al., 2016b; Liu et al., 2017; Liang et al., 2019; Li et al., 2020b). Spatial heterogeneity of single functions of single ecosystems has been proposed by ecologists and botanists at landscape and regional levels (Lin et al., 2010; Giese et al., 2013; Liu et al., 2018). Some scholars reported that morphological variation and spatial distribution of plant functional traits are the results of environmental filtering and biological interactions, reflecting plant adaptations to their habitats (Duran et al., 2019; Ma et al., 2019; Zhang et al., 2020b). Therefore, by analyzing the spatial distribution of functional traits and their relationship to environment, it is possible to how plants respond to environmental changes and how the responses affect the functions of single ecosystems.

Arid regions account for about 41% of the world’s total land, and about 38% of the population lives in arid regions (Reynolds et al., 2007). Due to the influences of climate change and anthropogenic disturbances, the aridification of terrestrial ecosystems is exacerbating (Gao and Giorgi, 2008; Feng and Fu, 2013). In Xinjiang, China, the desert area (65.46 × 10^4^ km^2^) accounts for 39% of the total area of Xinjiang, and has increased significantly (Ni and OuYang, 2006). Previous studies have shown that the proportions of C, N, and P in the total elemental content are relatively stable in desert ecosystems (Elser et al., 2000; Elser et al., 2010). However, plants with different life forms affect C, N, and P cycling to a certain extent, which could impact the spatial distribution of the functions of C, N, and P cycling in ecosystems (Wang and Yu, 2008; Wang et al., 2019).

Therefore, based on the spatial heterogeneity of ecosystem functions, the spatial distributions of the functions of C, N, and P cycling were predicted using the MDS of the dominant plant functional traits in a temperate desert region by regression kriging (RK), a method that combines regression modeling with kriging (Sarmadian et al., 2014; Pham et al., 2019). The objectives were to: (1) Select the functional traits of woody and herbaceous plants that play a dominant role in temperate desert ecosystems to construct the wMDS and hMDS, (2) determine the spatial distribution characteristics of C, N, and P cycling in temperate desert ecosystems using geostatistical methods, and (3) predict the spatial distribution characteristics of C, N and P cycling using linear and non-linear models based on the wMDS and hMDS. This study will advance our understanding of the relationship between plant functional traits and ecosystem function.

## 2 Materials and methods

### 2.1 Study site, sampling and experiment design

The study area is located in the Xinjiang Ebinur Lake Wetland National Nature Reserve on the southwestern edge of the Junggar Basin (44°30’ - 45°09’N, 82°36’ - 83°50’E). Surrounded by mountains on three sides, it is the lowest depression and water and salt enrichment centre (Wang et al., 2021a). In the Reserve, swamps, rivers, salt lakes, riparian forests, and deserts are the main landscapes. Aeolian sandy soil, grey brown desert soil, and grey desert soil are the zonal soils, and saline soil is the intrazonal soil. Central Asian and Mongolian flora is the main part of vegetation (He et al., 2014; Yang et al., 2014).

**Figure 1:**
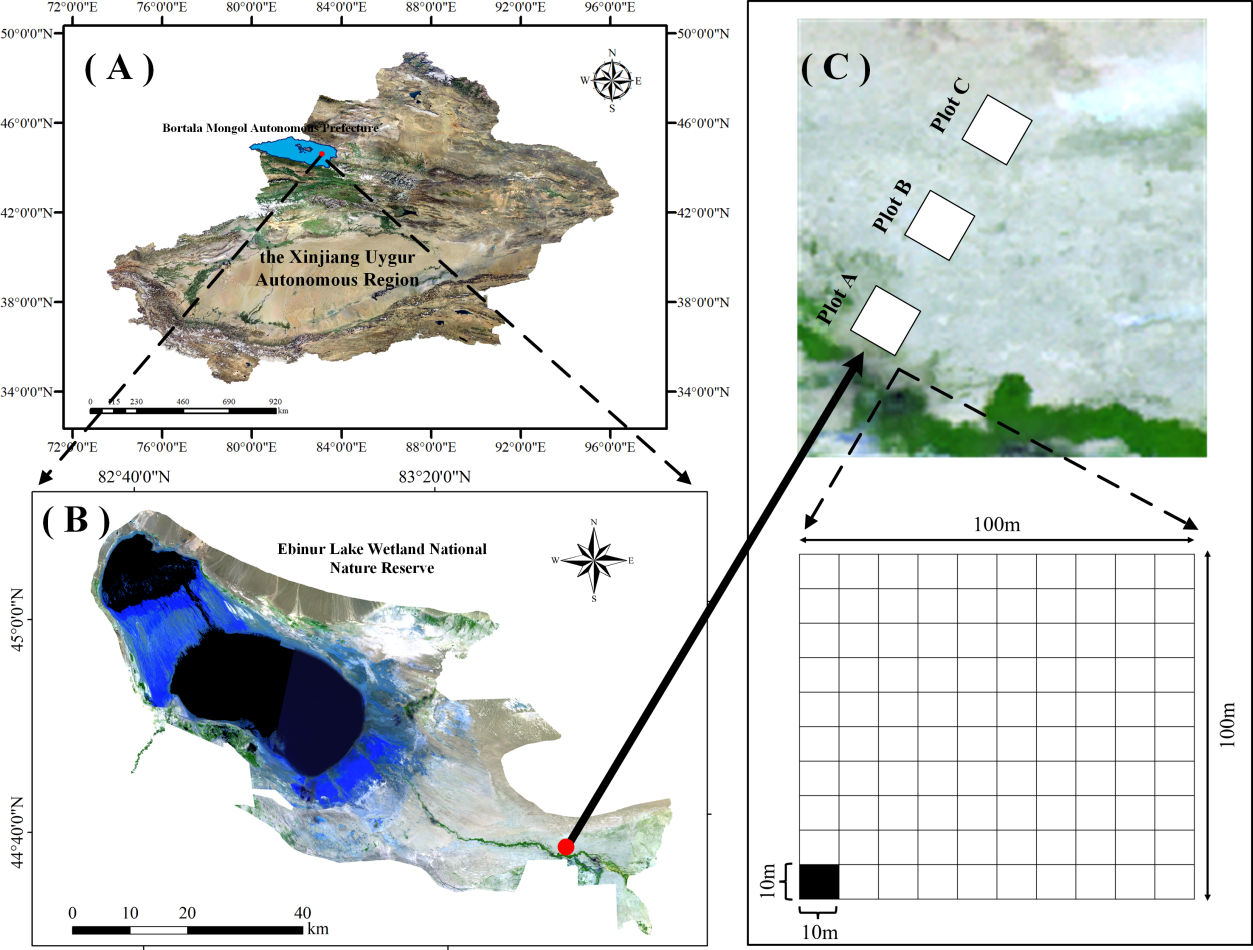
Overview map of the study area. **(A)** Ebinur Lake Basin, **(B)** the monitoring area within the basin, **(C)** and the sample sites. Plot A, Plot B, and Plot C represent river bank, transitional zone, and desert margin, respectively.

Three plots (100 m × 100 m) perpendicular to the Aqikesu River were set up from southwest to northeast (A-C) in the riparian forest-desert transition zone in the north of the Aqikesu River, and the distance between the plots was about 1.5 km (**Figure 1**). From July to August 2018, Herbaceous and woody plants were surveyed separately and the soil under the canopy of their collections was collected. Each plot was divided into 100 subplots (10 m × 10 m) (300 subplots totally), and the multiplicity, plant height (H), crown width (CW), leaf thickness (LT), leaf length (LL), leaf width (LW), DBH/basal diameter, leaf area (LA), and leaf fresh weight (LFW) of plants were recorded.

### 2.2 Plant and soil physicochemical experiments

Three plants of each species in each subplot were selected for the following determinations. The CW of trees was measured using a laser rangefinder (Dimetix-DAE-10-050, Dimetix, Switzerland), and that of shrubs and herbs was measured using a steel tape measure. For all trees in the subplots, the DBH was measured using a tape measure at a height of 1.3 m. For shrubs and herbs, the basal diameter was measured at 2.54 cm from the ground. Three to five leaves at each plant position (upper, middle, and bottom) were collected to determine the LT, LL, and LW using digital vernier caliper. To measure the LA, a 1 cm scale was marked on the lower right corner of a paper, then 10-20 leaves were laid flatly on the paper, followed by the photographing using a camera parallel to the paper. The pictures were processed using Photoshop software (2020CC, Adobe, USA) to obtain LA. After LA measurement, the leaves were transferred in sealed bags, weighed immediately (fresh weight), and dried for the measurements of dry weight and C, N, and P concentrations according to the methods of Bao (2000) (**Table S1**).

Within each subplot, five sampling points were selected along the diagonal, and the 0-30 cm soil layer was sampled at each sampling point for the determinations of soil C, N, and P concentrations (Bao, 2000) (**Table S1**).

### 2.3 Statistical analyses

#### 2.3.1 CWM

The CWM was calculated as a sample of the functional trait values within each subplot, based on the species diversity in each subplot and the measured functional trait values.


(1) $$\begin{array}{c} CWM=\frac{\sum_{i}^{n}A_{i}\times Trait_{i}}{\sum_{i}^{n}A_{i}}\; \end{array}$$

where $A_{i}$ is the species abundance in a subplot, and $Trait_{i}$ is the functional trait value of a species in a subplot (Ren, 2021).

#### 2.3.2 Construction of minimal data sets of plant functional traits

Sixteen plant functional traits were selected, including leaf carbon (LC), leaf nitrogen (LN), leaf phosphorus (LP), specific leaf area (SLA), specific leaf weight (SLW), LA, leaf dry matter content (LDMC), leaf water content (LWC), LFW, leaf dry weight (LDW), diameter at breast height (DBH) (stem base diameter (SBD)), H, LT, LL, LW, and width to length ratio (WLR) to perform Kaiser Mayer-Olkin (KMO) test and Bartlett’s test based on partial correlation. If tests were passed, factor analysis was performed on the selected traits. All above analyses were performed by using the *psych* and *vegan* package in R software (R Development Core Team, 2021).

The Norm value is an important reference for trait selection and MDS construction. The Norm value indicates the combined loading of a trait on multiple principal components with PC ≥ 1. So the larger the Norm value of a trait, the higher its combined loading value, and the more information on the principal components with PC ≥ 1 it has (Wu et al., 2019). Norm values were calculated as follows:


(2) $$\begin{array}{c} N_{ik}=\sqrt{\sum_{i=1}^{n}\left(U_{ik}^{2}\lambda_{k}\right)} \end{array}$$

where $N_{ik}$ is the combined loadings of the *i*th variable on *k* principal components with eigenvalue ≥ 1(Norm value), $U_{ik}$ is the loading of the *i*th variable on the *k*th principal component, and $\lambda_{k}$ is the eigenvalue of the *k*th principal component (Wu et al., 2019).

Based on the results of the factor analysis, principal component variables with eigenvalues greater than 1 (PC > 1) were screened out. Among the variables, the traits with factor loadings |PC| ≥ 0. 5 were screened out and grouped by different principal component variables. If a trait had a factor loading value |PC| ≥ 0.5 in two main variables, it was classified into the group with the lower correlation. Norm values of each group of traits were compared. Traits with Norm values in the top 10% in each group were retained, and the rest was discarded. If there were multiple traits in the top 10%, the correlations between the trait with the highest Norm value in each group and the traits in the top 10% were checked. If the correlation coefficient |r| ≥ 0.5, then the trait with higher Norm value and coefficient of variation was put into the MDS. If |r|< 0.5, then both were put into the MDS (Pulido et al., 2017).

#### 2.3.3 Minimum data set test model

Linear and non-linear scores of each trait was used to assign scores to the functional traits in the MDS:


(3) SL=XXMax

(4) SL=XMinX

where *S_L_* is the score derived from the linear score, ranging from 0 to 1, *X* is the value of a functional trait in the MDS, and *X_max_* and *X_min_* are the maximum and minimum value of each functional trait, respectively.

Equation (3) was applied to positive functional traits (the higher the value, the better the plant growth), while equation (4) was applied to negative functional traits (the lower the value, the better the plant growth) (Askari and Holden, 2014; Li et al., 2020a). The formula for the non-linear function:


(5) SNL=a1+(XXm)b

where *S_NL_* is the score derived from the non-linear score, ranging from 0 to 1, *a* is the maximum value that can be obtained for a functional trait, which is defined as 1 in this study, *X* is the value of a functional trait in the MDS, *X_m_* is the average value of a functional trait, and *b* is the slope, which is set to -2.5 for positive functional traits and +2.5 for negative functional traits (Zhang et al., 2011a; Raiesi, 2017).

After all functional traits in the MDS were assigned a score, the scores of all functional traits were summed using the following formula. The evaluation index *FTEI_A_* is the average of the scores of the traits in the MDS, while *FTEI_W_* is the sum of the scores of the functional traits multiplied by the corresponding weights (Askari and Holden, 2015).


(6) $$\begin{array}{c} FTEI_{A}=\sum_{i=1}^{n}S_{i}\times n^{-1} \end{array}$$

(7) $$\begin{array}{c} FTEI_{W}=\sum_{i=1}^{n}W_{i}\times S_{i} \end{array}$$

where *FTEI_A_* and *FTEI_W_* are the functional trait evaluation indices calculated without and with weights, respectively, *S_i_* is the score of the functional trait *i*, *n* is the number of functional traits in the MDS, and *W_i_* is the weight of the functional trait *i*. The weight was determined by the ratio of the characteristic roots of the principal component of functional trait *i* in the PCA analysis to the sum of all characteristic roots in the MDS (Guo et al., 2017; Jin et al., 2018).

#### 2.3.4 Ecosystem functions

The three ecosystem functions were the C, N, and P cycling in this study. The C cycling indicators included plant organic carbon and soil organic carbon, the N cycling indicators included soil nitrate nitrogen, ammonium nitrogen, and total nitrogen, and plant leaf total nitrogen, the P cycling indicators included soil available phosphorus and total phosphorus and plant leaf total phosphorus. All above were calculated by the average method (Maestre et al., 2012; Bowker et al., 2013).


(8) $$\begin{array}{c} EF=\frac{1}{n}\sum_{i=1}^{n}g\left(r_{i}\left(f_{i}\right)\right) \end{array}$$

where EF is a single ecosystem function, *f_i_* is the measured value of function *i*, *r_i_* is the mathematical function that converts *f_i_* to a positive value, *g* is the normalization of all measured values, and *n* represents the number of functions measured.

#### 2.3.5 Linear and non-linear predictive models

In this study, linear and non-linear models were used to predict the functions of single ecosystems based on the MDSs of plant functional traits. Models selected 70% sample size of C, N and P cycle indices of each first-level plot (100 m × 100 m) for training, and the remaining 30% sample size for model verification. The linear model was constructed by using partial least squares regression (PLS), which was based on covariance regression. PLS models can effectively catch the unique contributions of each independent variable to overcome multicollinearity. PLS models were constructed by the *pls* package in R software (Ge et al., 2018; Tan et al., 2020).

The non-linear model was constructed by using the Random Forest (RF) and BP neural network (BPNN). RF algorithm was based on the statistical learning theory of decision trees, which can effectively process high-dimensional data and overcome the overfitting. In this study, the RF model set the number of trees to 100, the GBM used the default setting, the maximum number of iterations was set to 500, the linear output unit was used, and the grid was set with the option to optimize the hyperparameters. The model constructions using the RF were completed by using the *random Forest* package (Breiman, 2001; Poggio et al., 2019; Vilchez-Mendoza et al., 2022). BPNN model is a multi-layer feed-forward neural network that continuously approximates the desired output based on the backward transmission of errors to obtain a prediction. In this study, the BPNN model built four hidden layers with five nodes in each layer and used backprop algorithm for calculation. The model constructions using the BPNN were completed by using the *neural net* package (Huang et al., 2019a; Morais et al., 2021).

Root mean square error (RMSE) and mean absolute error (MAE) were used to test the accuracy of the predictive models (Qiu et al., 2010; Guo et al., 2013; Knadel et al., 2021).


(9) $$\begin{array}{c} RMSE=\sqrt{\frac{1}{N}\sum_{i=1}^{N}{\left(observed_{i}-predicted_{i}\right)}^{2}} \end{array}$$

(10) $$\begin{array}{c} MAE=\frac{1}{N}\sum_{i=1}^{N}\left|\left(observed_{i}-predicted_{i}\right)\right| \end{array}$$

where $predicted_{t}$ is the predicted value of a subplot, $observed_{t}$ is the measured value of a subplot, and *N* is the number of subplots. The smaller the RMSE and MAE, the higher the prediction accuracy.

#### 2.3.6 Geostatistical analysis

##### 2.3.6.1 Regression kriging

Regression kriging (RK) is a spatial interpolation technique that performs kriging interpolation on the prediction residuals by combining the regression of the dependent variable on the predictor variables (such as environmental variables) (Hengl et al., 2004). That is to say, RK is a hybrid method that combines a simple or multiple linear regression model with ordinary kriging for predicting residuals. RK allows the auxiliary variables to interpolate the dependent variables at unsampled locations (Hengl et al., 2007). In this study, Partial least squares kriging (PLSK) (Guo et al., 2021), Random forest kriging (RFK) (Breiman, 2001; Behnamian et al., 2017), and Back propagation neural network (BPNNK) models were constructed (Li et al., 2017; Chen et al., 2020). Regression Kriging (RK)was constructed using the predicted values from the PLS, RF and BPNN models in combination with the Ordinary Kriging (OK) method. Aim of this method was to establish linear and non-linear mapping relationships between the MDSs and a single ecosystem function by PLS, RF, and BPNN. Relative coordinates were established in each subplot, and then the residual terms were spatially interpolated using the OK method to obtain the final prediction results. This was completed by using the *automap* and *gstat* packages (Meng and Liu, 2013; Mukherjee et al., 2015).


(11) $$\begin{array}{l} \hat{z}\left(s_{o}\right)=\hat{m}\left(s_{o}\right)+\hat{e}\left(s_{o}\right)\\ \begin{array}{c} =\sum_{k=0}^{p}{\hat{\beta }}_{k}\cdot q_{k}\left(s_{o}\right)+\sum_{i=0}^{n}{\hat{\lambda }}_{i}\cdot e\left(s_{i}\right) \end{array} \end{array}$$

where $\hat{z}\left(s_{o}\right)$ is the interpolation result at the predicted subplot $s_{o}$ , $\sum_{k=0}^{p}{\hat{\beta }}_{k}\cdot q_{k}\left(s_{o}\right)$ is the deterministic part of the fitting by regression, $\sum_{i=0}^{n}{\hat{\lambda }}_{i}\cdot e\left(s_{i}\right)$ is the interpolation result on the regression residuals by OK method, *k* is the position number in the fitting by regression, *p* is the sample size of the regression model based on the predicted values, ${\hat{\beta }}_{k}$ is the coefficient of the regression model, ${\hat{\beta }}_{0}$ is the intercept when *k*=0, *i* is the position number at the regression residual interpolation, *n* is the sample size for kriging interpolation of the residual value based on the predicted values, $q_{k}\left(s_{o}\right)$ is the value of the auxiliary variable at the predicted position $s_{o}$ , ${\hat{\lambda }}_{i}$ is the OK interpolation weight determined by the spatial correlation structure of the regression residual, and $e\left(s_{i}\right)$ is the residual at position $s_{i}$ .

##### 2.3.6.2 Semi-variable functions

The traits were tested for normal distribution before geostatistical analysis. If the traits did not follow normal distribution, they would be transformed with Box-Cox (Wang et al., 2021a). The spatial variability of ecosystem functional diversity was analyzed using geostatistical software (GS+, version 9.0, Gamma Design Software. LLC, USA), and a semi-covariance function model was fitted. By analyzing the nugget (*C*_0_), structural variance (*C*), sill (*C*_0_ *+ C*), variation range (Range), and nugget-sill ratio (*C*_0_ */(C*_0_ *+ C)*), the proportion of nugget variance in total spatial heterogeneity variance was determined, i.e. the proportion of structural variance in the total variance. It is often used to describe the degree of variation in the spatial heterogeneity of study objects. If *C*_0_ */(C*_0_ *+ C)*< 25%, the variable has strong spatial autocorrelation; if *C*_0_ */(C*_0_ *+ C)* is between 25% and 75%, the variable has moderate spatial autocorrelation among the variables; if *C*_0_ */(C*_0_ *+ C)* > 75%, the variable has weak spatial autocorrelation (Robertson et al., 1993; Zartman, 2005).


(12) $$\begin{array}{c} \gamma \left(h\right)=\frac{1}{2N\left(h\right)}\sum_{i=1}^{N\left(h\right)}{\left[A_{i}\left(x_{i}\right)-A_{i}\left(x_{i}+h\right)\right]}^{2} \end{array}$$

where *γ(h)* is the semi-variance of the interval class *h*, *N(h)* is the number of samples separated by the lag distance, and *Ai(xi)* and *Ai(xi+h)* are the measurement variables for spatial locations *i* and *i+h*, respectively. There are four types of models: linear, spherical, exponential, and Gaussian. The coefficient of determination (*R^2^*) and the residual sum of squares (*RSS*) were used to select the best-fitting model. The larger the *R^2^*, the smaller the *RSS*, the better the model fitted (Cambardella et al., 1994; Wang et al., 2021b).

## 3 Results

### 3.1 Indicators of minimum data set of functional traits

In the three plots (A-C), where were the species and frequencies of plant in **Table S2**, the wMDS and hMDS were constructed based on the functional traits of woody and herbaceous plants in the subplots (**Table 1**). The results showed that the SBD, H, LT, LL, LW, and WLR of herbaceous plants in plot C were different (*P*< 0.05) from those of herbaceous plants in plots A and B. The SBD and H in plot B was different (*P*< 0.05) from those in plot A. Therefore, the functional traits of woody and herbaceous plants in the three plots were combined to construct wMDS and hMDS.

**Table 1:** Analysis of variance for functional traits of woody and herbaceous plants in plots A, B and C.

Woody plant				Herbaceous plant			
Trait variables	Plot A	Plot B	Plot C	Trait variables	Plot A	Plot B	Plot C
LC	0.992 a	0.990 a	0.989 a	LC	0.992 a	0.978 a	0.983 a
LN	0.991 a	0.991 a	0.987 a	LN	0.990 a	0.987 a	0.981 a
LP	0.991 a	0.988 a	0.981 a	LP	0.990 a	0.982 a	0.969 a
SLA	0.985 a	0.974 a	0.966 a	SLA	0.975 a	0.972 a	0.980 a
SLW	0.984 a	0.971 a	0.963 a	SLW	0.966 a	0.962 a	0.978 a
LA	0.893 a	0.944 a	0.924 a	LA	0.938 a	0.836 a	0.924 a
LDMC	0.988 a	0.983 a	0.985 a	LDMC	0.993 a	0.948 a	0.962 a
LWC	0.993 a	0.992 a	0.991 a	LWC	0.994 a	0.983 a	0.991 a
LFW	0.896 a	0.950 a	0.926 a	LFW	0.955 a	0.890 a	0.926 a
LDW	0.898 a	0.948 a	0.927 a	LDW	0.950 a	0.840 a	0.917 a
DBH	0.658 a	0.763 a	0.797 a	SBD	0.593 a	0.879 b	0.629 b
H	0.928 a	0.855 a	0.885 a	H	0.982 a	0.847 ab	0.921 b
LT	0.827 a	0.905 a	0.956 a	LT	0.983 a	0.846 a	0.965 b
LL	0.844 a	0.833 a	0.747 a	LL	0.966 a	0.803 a	0.964 b
LW	0.274 a	0.845 a	0.878 a	LW	0.485 a	0.856 a	0.933 b
WLR	0.247 a	0.913 a	0.858 a	WLR	0.538 a	0.887 a	0.937 b

The KMO test based on partial correlation on the CWMs of the 16 plant functional traits showed that the KMO value of woody plants was 0.77 (*P*< 0.001), with Bartlett’s test *P*< 1.7e-16, and that of herbaceous plants was 0.74 (*P*< 0.001), with Bartlett’s test *P*< 2.2e-16. This indicates that there is a correlation between the woody and herbaceous functional traits, and it is suitable for factor analysis.

**Figure 2:**
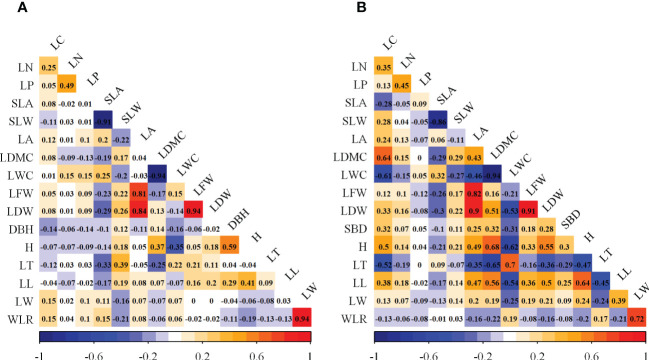
Pearson correlation analysis of functional traits in woody and herbaceous communities. **(A)** Woody functional trait indicators; **(B)** Herbaceous functional trait indicators.

Based on the results of the factor analysis (**Table 2**), the CWMs of the 16 woody plant functional traits were initially divided into seven groups. For group 1, because the ratios of the Norm values of LC, LN, LP, and LL to that of H were less than 90%, H was included in the wMDS. For group 2, the ratio of the Norm value of SLW to that of SLA was higher than 90%, and the Pearson correlation analysis (**Figure 2A**) showed that the correlation coefficient was greater than 0.5, thus SLA was included in the wMDS. With the same screening method as group 2, the LDW, LWC, and LW were included into the wMDS for groups 3, 4, and 7, respectively. Group 5 and 6 only had DBH and LT, respectively, which were included into the wMDS. Therefore, seven traits (H, SLA, LDW, LWC, LW, DBH, and LT) were ultimately included in the wMDS.

**Table 2:** Factor loading matrices, groupings and Norm values for functional traits in woody plant communities.

Trait variables	Principal component (PC)						Groups	Norm value	MDS
	PC1	PC2	PC3	PC4	PC5	PC6			
LC	0.062	-0.055	-0.032	0.094	-0.063	-0.051	1	0.218	―
LN	0.003	0.027	0.074	0.009	-0.010	0.009	1	0.119	―
LP	0.065	0.005	0.081	0.063	-0.062	0.007	1	0.201	―
SLA	-0.070	-0.957	0.133	0.053	-0.031	-0.110	2	1.620	Enter
SLW	0.051	0.945	-0.091	-0.107	0.037	0.163	2	1.602	―
LA	0.937	-0.280	-0.064	0.044	-0.045	-0.022	3	1.738	―
LDMC	-0.004	0.083	-0.966	-0.035	0.036	-0.100	4	1.439	―
LWC	-0.005	-0.127	0.966	0.023	-0.055	0.086	4	1.446	Enter
LFW	0.951	0.181	0.175	-0.011	-0.013	0.081	3	1.744	―
LDW	0.960	0.207	-0.108	-0.005	-0.004	0.023	3	1.753	Enter
DBH	-0.054	0.053	-0.072	-0.033	0.949	0.020	5	1.186	Enter
H	0.073	0.072	-0.243	-0.074	0.343	-0.023	1	0.590	Enter
LT	0.064	0.246	0.177	-0.059	0.019	0.946	6	1.220	Enter
LL	0.102	0.101	-0.023	-0.019	0.128	0.031	1	0.299	―
LW	0.013	-0.056	0.043	0.982	-0.004	-0.012	7	1.294	Enter
WLR	0.009	-0.090	0.013	0.973	-0.038	-0.052	7	1.288	―
Characteristic value	3.174	2.780	2.177	1.722	1.525	1.377	―	―	―
Variance contribution rate of the PC (%)	17.144	12.992	12.806	12.205	6.586	6.044	―	―	―
Cumulative contribution rate of the PC(%)	17.144	30.136	42.941	55.146	61.732	67.776	―	―	―

The CWMs of herbaceous plant functional traits were divided into six groups (**Table 3**). For group 1, the ratio of the Norm values of LC, LN, LP, SBD, and LT to that of H was lower than 90%, then H was included in the hMDS. For group 2, the ratio of the Norm value of SLW to that of SLA was higher than 90% and the Pearson correlation analysis (**Figure 2B**) showed that the correlation coefficient was greater than 0.5, then SLA was included in the hMDS. With the same screening method as group 2, LFW, LWC, and LW were included in the hMDS for groups 3, 4, and 6, respectively. Group 5 had LL only. Therefore, six traits (H, SLA, LFW, LWC, LW, and LL) were ultimately included in the hMDS

**Table 3:** Factor loading matrix, groupings and Norm values for functional traits in herbaceous communities.

Trait variables	Principal component (PC)					Groups	Norm value	MDS
	PC1	PC2	PC3	PC4	PC5			
LC	0.086	0.390	0.165	-0.010	0.097	1	0.638	―
LN	0.077	0.055	0.020	0.005	0.059	1	0.213	―
LP	-0.074	-0.015	-0.048	-0.054	-0.010	1	0.201	―
SLA	-0.103	-0.131	-0.943	-0.027	-0.037	2	1.341	Enter
SLW	0.022	0.110	0.953	0.047	0.026	2	1.330	―
LA	0.899	0.265	-0.194	0.005	0.125	3	2.189	―
LDMC	0.170	0.882	0.149	-0.034	0.173	4	1.335	―
LWC	-0.210	-0.884	-0.155	-0.006	-0.153	4	1.367	Enter
LFW	0.965	-0.061	0.157	0.025	0.087	3	2.307	Enter
LDW	0.911	0.276	0.162	-0.007	0.116	3	2.214	―
SBD	0.132	0.142	0.044	-0.003	0.062	1	0.384	―
H	0.289	0.400	0.096	-0.004	0.269	1	0.951	Enter
LT	-0.136	-0.461	0.014	-0.016	-0.141	1	0.743	―
LL	0.278	0.297	0.059	0.047	0.867	5	1.284	Enter
LW	-0.094	-0.13	0.015	0.944	-0.185	6	1.249	Enter
WLR	0.130	0.124	0.065	0.905	0.274	6	1.246	―
Characteristic value	5.638	1.971	1.913	1.604	1.370	―	―	―
Variance contribution rate of the PC (%)	18.098	15.056	12.387	10.757	6.634	―	―	―
Cumulative contribution rate of the PC (%)	18.098	33.154	45.541	56.298	62.932	―	―	―

### 3.2 Minimum data set test

**Figure 3:**
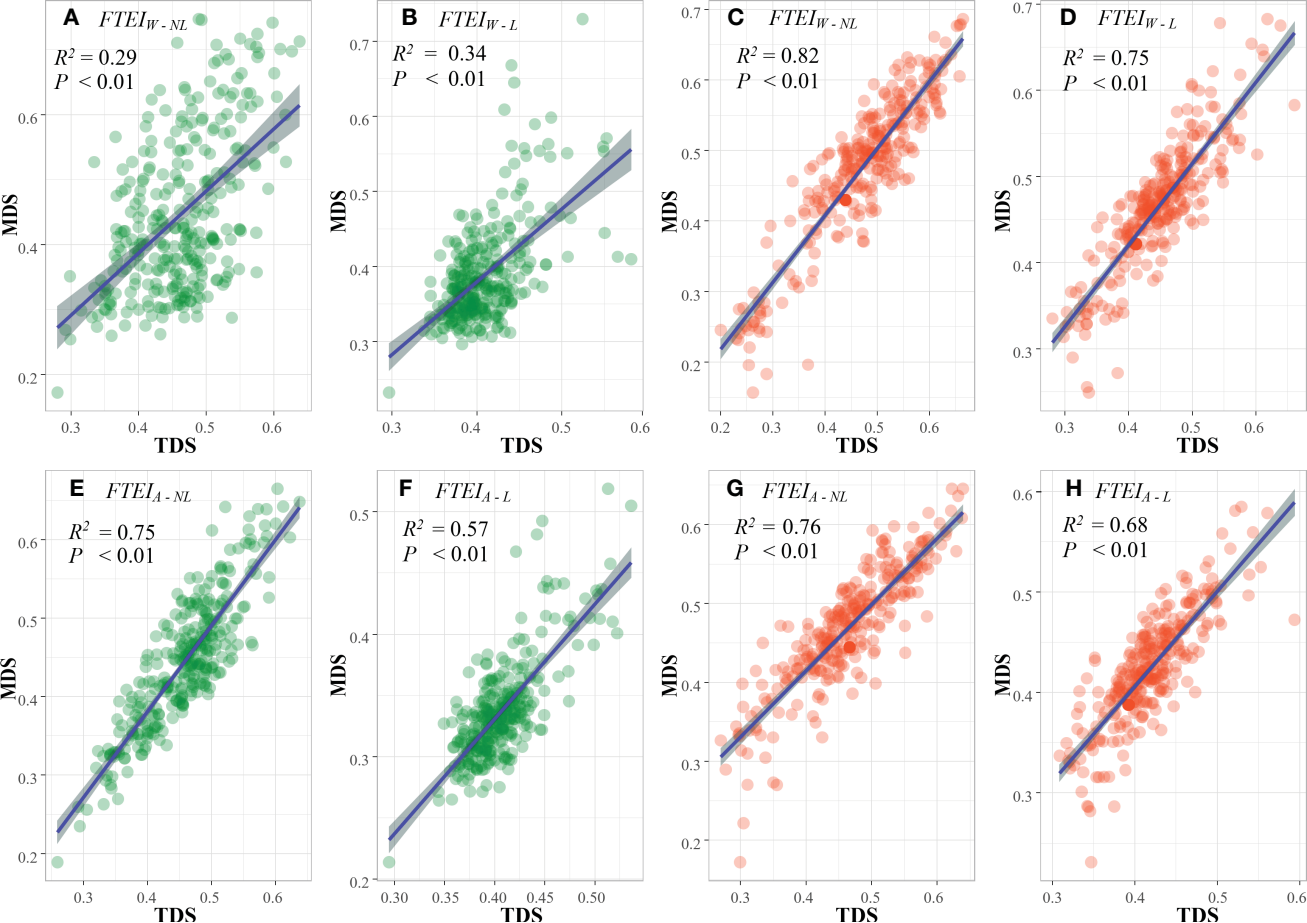
Regression analysis of four cross-tests for MDS and TDS. **(A, B, E, F)** are MDS tests for woody functional traits; **(C, D, G, H)** are MDS tests for herbaceous functional traits.

Four cross-validation methods including *FTEI_W - L_* (linear weight evaluation method), *FTEI_A - L_* (linear average evaluation method), *FTEI_W - NL_* (non-linear weight evaluation method), and *FTEI_A - NL_* (non-linear average evaluation method) were used to test the correlation between MDS and TDS in this study. The *R^2^* of the linear regression of *FTEI_W - L_*, *FTEI_A - L_*, *FTEI_W - NL_*, and *FTEI_A - NL_* of MDS and TDS of woody plants were 0.29, 0.34, 0.75, and 0.57, respectively, and those of herbaceous plants were 0.82, 0.75, 0.76, and 0.68, respectively. Overall, the test results by *FTEI_W - L_*, *FTEI_A - L_*, *FTEI_W - NL_*, and *FTEI_A - NL_* all showed significant correlation (**Figure 3**).

In this study, the *S_L_* method outperformed the *S_NL_* method when the same evaluation system was used (**Figure 3**). By comparing different traits, it was found that the correlation coefficients calculated by *FTEI_A_* were higher than those calculated by *FTEI_W_* under the same assignment function method. Furthermore, MDS and TDS showed a positive correlation (*P<* 0.001) in all the resultant models tested by cross-validation. Therefore, the constructed wMDS and hMDS can replace TDS.

### 3.3 Spatial distribution characteristics of ecosystem functions

**Figure 4:**
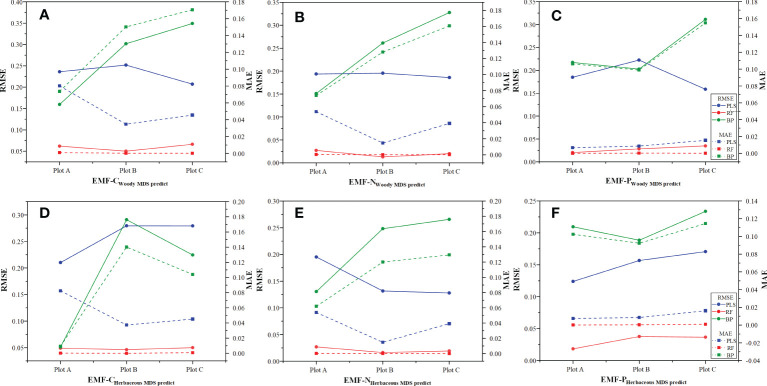
Prediction model accuracy tests. **(A–C)** are ecosystem functions predicted by MDS for woody functional traits; **(D–F)** are ecosystem functions predicted by MDS for herbaceous functional traits.

The optimal semi-variance function model for predicting C cycling based on the raw values, wMDS, and hMDS was the exponential model. The analysis results of *C*_0_ */(C*_0_ *+ C)* showed that the structure of spatial variability had strong spatial autocorrelation (**Table 4**). This suggests that the spatial distribution of C cycling is mainly influenced by structural factors. The optimal semi-variance models based on raw and predicted values of N cycling were exponential and Gaussian models (**Table 5**). Except for the moderate spatial autocorrelation of the PLS model-predicted values based on the wMDS in plot B, the *C*_0_ */(C*_0_ *+ C)* of the remaining raw and predicted values also showed strong spatial autocorrelation. This indicates that there is an error in PLS prediction accuracy and the spatial heterogeneity is dominated by structural factors. The optimal semi-variance models based on raw and predicted values of P cycling were exponential and Gaussian models (**Table 6**). The *C*_0_ */(C*_0_ *+ C)* of raw values and predicted values based on wMDS for plot B showed moderate spatial autocorrelation, while that of the remaining values showed strong spatial autocorrelation, with structural factors dominating spatial heterogeneity. In summary, the C, N, and P cycling showed strong spatial autocorrelation, and the prediction accuracy of the RF and BPNN models were better than that of PLS model. By comparing the C, N, and P cycling predicted results of the three models based on the wMDS, it was found that the prediction accuracy of the RF model was higher than that of the BPNN and PLS models (**Figures 4A–C**). Furthermore, the same results were obtained by comparing the predicted results of the three models based on the hMDS (**Figures 4D–F**).

**Table 4:** Statistical parameters of the ecosystem C cycle function.

(Woody) Plots/Kriging		Models	C_0_	C_0_+C	C_0_/(C_0_+C) (%)	Range (m)	R^2^	RSS
A	Real	Exp	0.0044	0.0783	5.6	5.6	0.614	3.70E-05
	PLS	Exp	0.0056	0.0932	6.0	3.4	0.011	2.88E-04
	RF	Exp	0.0009	0.0284	3.3	4.5	0.313	5.75E-06
	BP	Exp	0.0016	0.0336	4.8	5.8	0.667	6.31E-06
B	Real	Exp	0.0014	0.0620	2.3	10.7	0.913	2.25E-05
	PLS	Exp	0.1700	0.4950	34.3	62.7	0.978	2.39E-04
	RF	Exp	0.0120	0.0395	30.4	40.4	0.988	1.30E-06
	BP	Exp	0.0016	0.0492	3.3	11.2	0.863	2.40E-05
C	Real	Exp	0.0003	0.0760	0.4	3.3	0.009	1.97E-04
	PLS	Exp	0.0070	0.1300	5.4	6.8	0.749	9.45E-05
	RF	Exp	0.0014	0.0282	5.0	3.6	0.013	3.04E-05
	BP	Exp	0.0006	0.0213	2.7	4.4	0.066	1.26E-05
(Herbaceous) Plots/Kriging		Models	*C*_0_	*C*_0_ *+C*	*C*_0_ */(C*_0_ *+C)* (%)	Range (m)	*R^2^*	*RSS*
A	Real	Exp	0.0067	0.0500	13.4	10.2	0.904	1.21E-05
	PLS	Exp	0.0040	0.0415	9.6	5.5	0.601	8.37E-06
	RF	Exp	0.0023	0.0169	13.7	9.1	0.895	1.19E-06
	BP	Exp	0.0035	0.0251	14.1	7.1	0.836	2.25E-06
B	Real	Exp	0.0079	0.1008	7.8	6.5	0.989	1.90E-06
	PLS	Exp	0.0860	0.5910	14.6	16.6	0.997	1.10E-04
	RF	Exp	0.0093	0.0723	12.9	9.4	0.985	3.46E-06
	BP	Exp	0.0053	0.0397	13.4	10.7	0.977	1.99E-06
C	Real	Exp	0.0004	0.0681	0.6	4.7	0.269	7.42E-05
	PLS	Exp	0.0240	0.4460	5.4	4.4	0.427	1.02E-03
	RF	Exp	0.0014	0.0379	3.7	5.0	0.386	1.88E-05
	BP	Exp	0.0000	0.0147	0.1	4.3	0.167	4.56E-06

**Table 5:** Statistical parameters of the ecosystem N cycle function.

(Woody) Plots/Kriging		Models	C_0_	C_0_+C	C_0_/(C_0_+C) (%)	Range (m)	R^2^	RSS
A	Real	Exp	0.0017	0.0162	10.4	5.7	0.759	7.11E-07
	PLS	Exp	0.0026	0.1112	2.3	8.7	0.505	3.59E-04
	RF	Exp	0.0006	0.0059	9.3	5.6	0.659	1.37E-07
	BP	Exp	0.0040	0.0353	11.5	5.8	0.798	2.96E-06
B	Real	Gau	0.0007	0.0044	16.3	10.1	0.936	4.41E-08
	PLS	Gau	0.0851	0.2252	37.8	41.7	0.998	1.71E-05
	RF	Gau	0.0003	0.0019	16.1	12.7	0.963	1.16E-08
	BP	Gau	0.0040	0.0294	13.6	10.1	0.968	1.01E-06
C	Real	Exp	0.0010	0.0087	11.6	6.4	0.807	2.24E-07
	PLS	Exp	0.0028	0.1236	2.3	0.3	0.001	1.23E-04
	RF	Exp	0.0005	0.0033	14.0	9.0	0.889	4.45E-08
	BP	Exp	0.0058	0.0196	11.7	6.5	0.800	7.89E-06
(Herbaceous) Plots/Kriging		Models	*C*_0_	*C*_0_ *+C*	*C*_0_ */(C*_0_ *+C)* (%)	Range (m)	*R^2^*	*RSS*
A	Real	Exp	0.0016	0.0157	10.2	5.9	0.908	2.63E-07
	PLS	Exp	0.0097	0.1224	7.9	2.5	0.092	8.77E-06
	RF	Exp	0.0004	0.0059	7.0	6.0	0.801	1.03E-07
	BP	Exp	0.0038	0.0386	9.8	6.3	0.902	2.04E-06
B	Real	Gau	0.0005	0.0047	11.4	6.5	0.118	1.30E-07
	PLS	Gau	0.0074	0.0737	10.0	9.0	0.752	3.28E-05
	RF	Gau	0.0002	0.0015	10.5	7.4	0.298	1.78E-08
	BP	Gau	0.0033	0.0354	9.3	5.7	0.030	4.72E-06
C	Real	Gau	0.0008	0.0070	11.6	6.3	0.099	2.03E-07
	PLS	Gau	0.0045	0.0425	10.6	7.2	0.470	5.70E-06
	RF	Gau	0.0003	0.0022	11.0	7.2	0.573	9.15E-09
	BP	Gau	0.0031	0.0363	8.5	1.8	0.001	5.45E-06

**Table 6:** Statistical parameters of the ecosystem P cycle function.

(Woody) Plots/Kriging		Models	C_0_	C_0_+C	C_0_/(C_0_+C) (%)	Range (m)	R^2^	RSS
A	Real	Gau	0.0006	0.0080	6.8	1.8	0.001	2.38E-07
	PLS	Gau	0.0159	0.1598	9.9	1.9	0.001	1.49E-04
	RF	Gau	0.0001	0.0028	4.8	1.9	0.001	2.42E-08
	BP	Gau	0.0023	0.0310	7.5	1.8	0.001	5.63E-06
B	Real	Gau	0.0107	0.0263	40.5	30.3	0.998	1.98E-07
	PLS	Gau	0.1285	0.3470	37.0	45.8	0.988	1.76E-04
	RF	Gau	0.0041	0.0130	31.2	29.5	0.999	1.01E-08
	BP	Gau	0.0259	0.0606	42.7	29.2	0.998	8.83E-07
C	Real	Gau	0.0009	0.0221	3.9	1.9	0.001	3.89E-06
	PLS	Gau	0.0071	0.1062	6.7	7.8	0.272	1.77E-04
	RF	Gau	0.0004	0.0081	4.3	8.3	0.362	1.31E-06
	BP	Gau	0.0015	0.0292	5.2	1.9	0.001	5.31E-06
(Herbaceous) Plots/Kriging		Models	*C*_0_	*C*_0_ *+C*	*C*_0_ */(C*_0_ *+C)* (%)	Range (m)	*R^2^*	*RSS*
A	Real	Gau	0.0006	0.0064	8.9	5.5	0.002	1.09E-06
	PLS	Gau	0.0107	0.0713	15	9.8	0.837	2.80E-05
	RF	Gau	0.0002	0.0022	10.4	6.1	0.013	1.20E-07
	BP	Gau	0.0015	0.0255	5.8	1.9	0.001	1.77E-05
B	Real	Exp	0.0053	0.0267	19.7	16.4	0.956	2.66E-06
	PLS	Exp	0.0194	0.1248	15.5	16.0	0.992	1.53E-05
	RF	Exp	0.0007	0.0085	8.7	12.9	0.936	4.41E-07
	BP	Exp	0.0062	0.0427	14.5	11.0	0.894	1.25E-05
C	Real	Gau	0.0028	0.0251	11.2	10.3	0.823	5.93E-06
	PLS	Gau	0.0102	0.1294	7.9	7.9	0.486	1.17E-04
	RF	Gau	0.0011	0.0083	12.8	10.2	0.802	6.61E-07
	BP	Gau	0.0075	0.0555	13.5	10.7	0.923	1.26E-05

**Figure 5:**
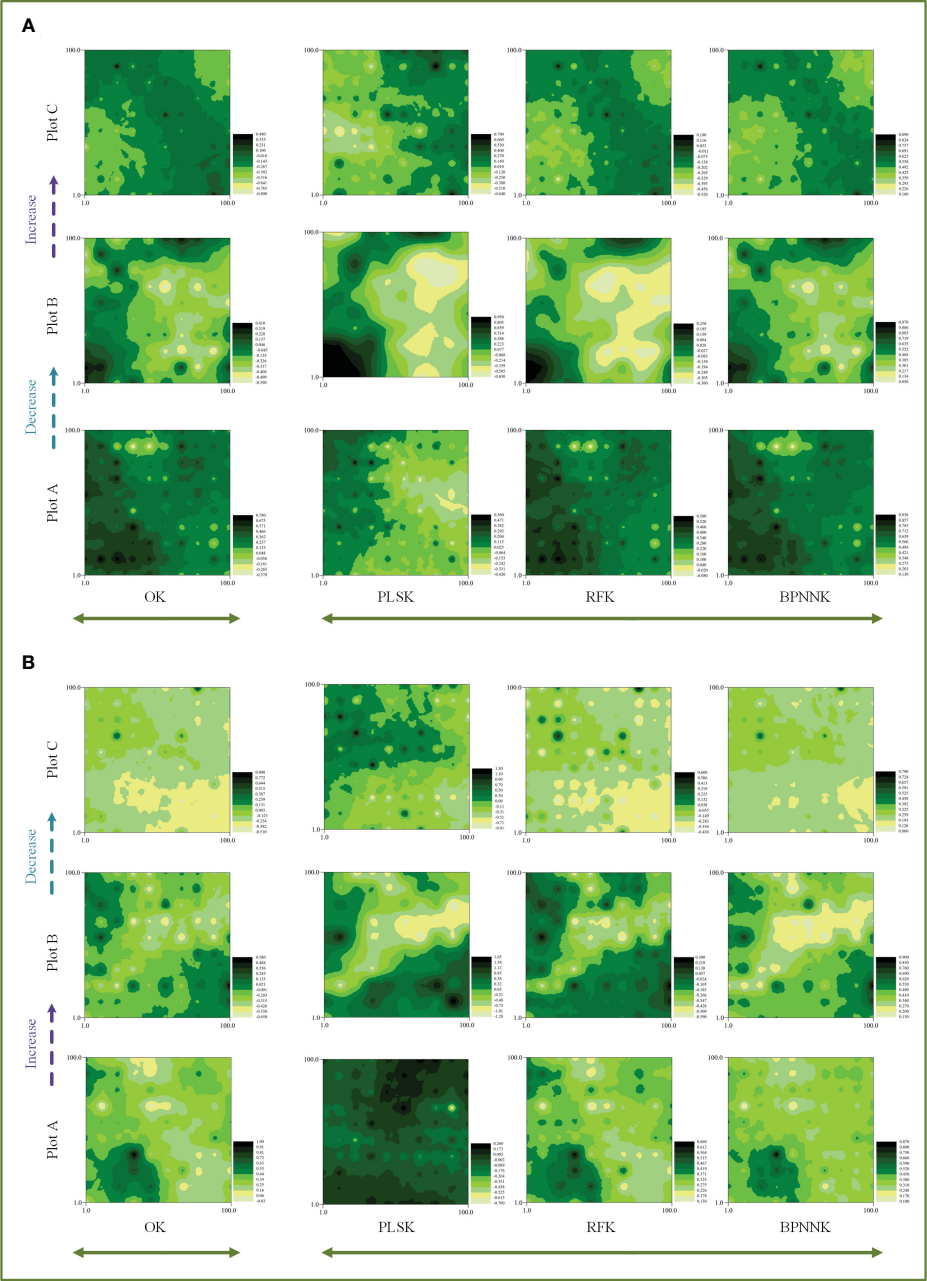
Spatial distribution characteristics of the carbon cycle, OK, Ordinary Kriging. **(A, B)** are the spatial distribution characteristics of carbon cycle prediction of woody MDS and herbaceous MDS respectively, PLSK, Partial least squares Kriging; RFK, Random forest Krieger; BPNNK, BP neural network Kriging.

**Figure 6:**
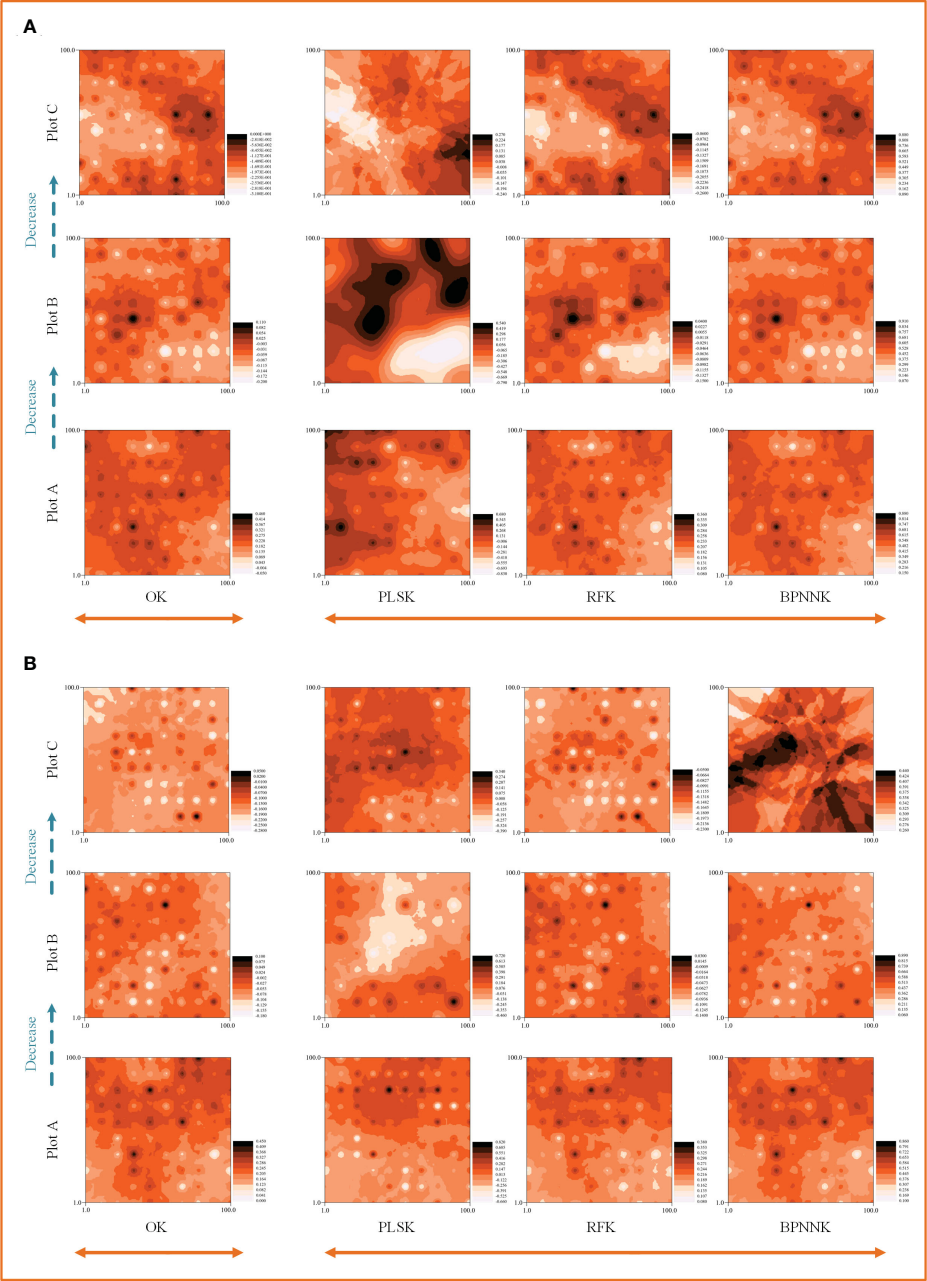
Spatial distribution characteristics of the nitrogen cycle, OK, Ordinary Kriging. **(A, B)** are the spatial distribution characteristics of carbon cycle prediction of woody MDS and herbaceous MDS respectively, PLSK, Partial least squares Kriging; RFK, Random forest Krieger; BPNNK, BP neural network Kriging.

**Figure 7:**
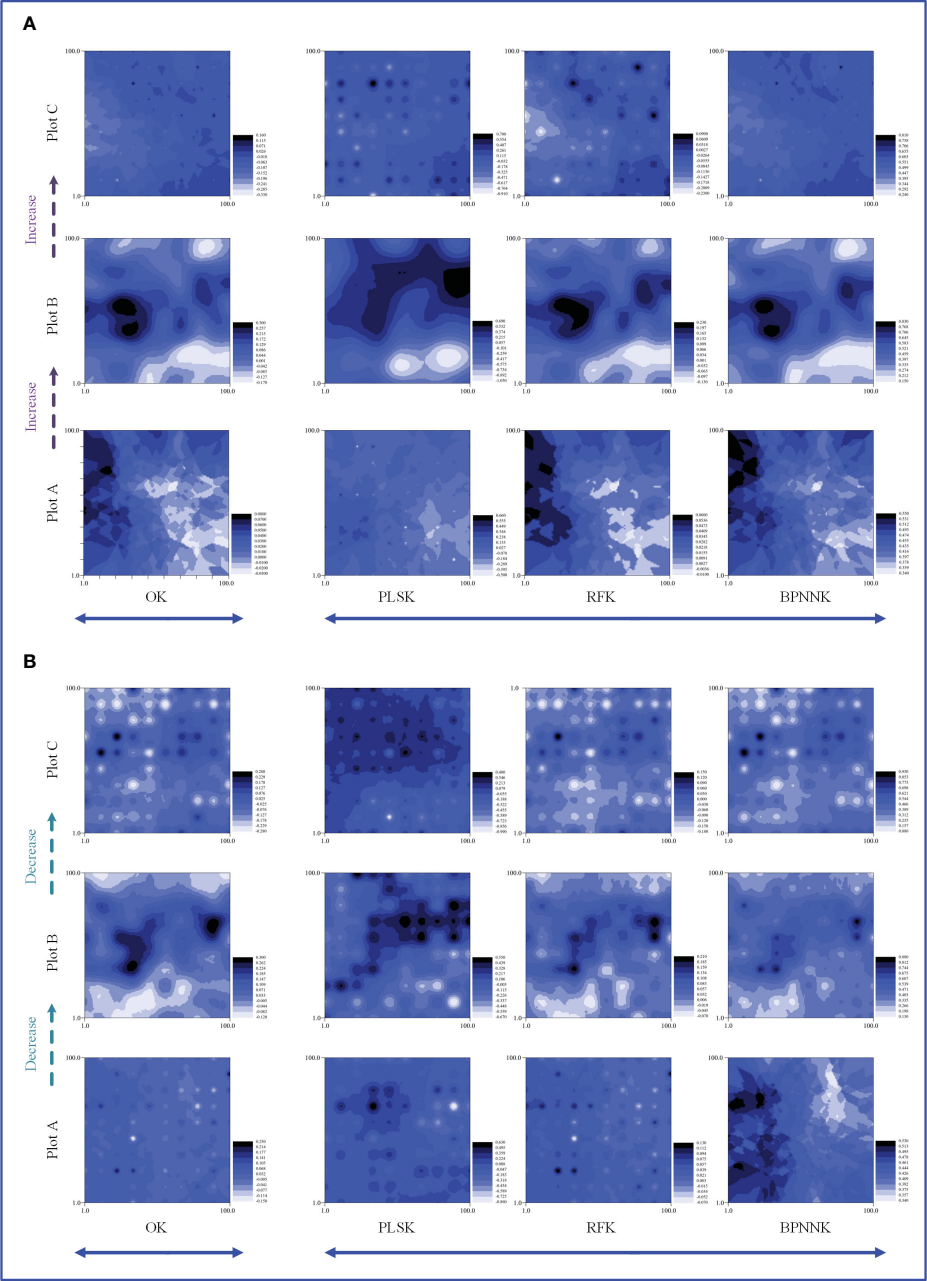
Spatial distribution characteristics of the phosphorus cycle, OK, Ordinary Kriging. **(A, B)** are the spatial distribution characteristics of carbon cycle prediction of woody MDS and herbaceous MDS respectively, PLSK, Partial least squares Kriging; RFK, Random forest Krieger; BPNNK, BP neural network Kriging.

The OK results showed that the ecosystem function C, N and P cycles from river bank to desert margin (Plot A-C). The C cycle of woody plants firstly decreased and then increased with gradients (**Figure 5**), while the carbon cycle of herbaceous plants was the opposite. The N cycles in both woody and herbaceous plants were weakened along the gradients (**Figure 6**). The P cycle of herbaceous plants was continuously decreased while the woody plants were continuously increased (**Figure 7**).

### 3.4 Regression kriging prediction

The RK visualization results based on wMDS are shown in (**Figures 5**, **6**, **7A**). The visualization results based on raw and predicted values all showed that the prediction accuracy of the BPNNK model was the highest, followed by RFK and PLSK models. The OK and RK visualization results of P cycling in plot B all showed “bull’s eye phenomenon”. The visualization results by hMDS (**Figures 5**, **6**, **7B**) based on raw and predicted values all showed that the prediction accuracy of the BPNNK model was similar to that of the RFK model, and the prediction accuracy of the PLSK model was the lowest. Furthermore, local extremum or over smoothing (lack of details) were shown in the P cycling in plot A, the C cycling in plot B, and the N cycling in plot C. Therefore, RF and BPNN are better than PLS in RK prediction. The accuracy test results also showed that RF and BPNN were better than PLS (**Figure 4**). Moreover, the RK visualization results based on wMDS were better than those based on hMDS (**Figures 5**–**7**).

## 4 Discussion

### 4.1 Minimum data set of functional traits

In this study, plant functional traits were used as predictor variables, and hMDS and wMDS were constructed by selecting important plant functional traits to predict ecosystem functions. According to the study results, morphological traits including SLA, DBH, PH, LW, and LT, and physiological traits including LWC and LDW were included in the wMDS. Previous studies have shown that plant leaf morphological traits could determine plant photosynthetic capacity (Cornelissen et al., 2003; Yu et al., 2014; Liu and Liang, 2016). Some scholars also reported that H and SBD (DBH) could reflect the adaptability of plants to environmental changes and their ability to acquire resources, and the length and thickness of the stems of woody plants were more sensitive to environmental filtering (Fernández et al., 2002; Liu et al., 2015). In this study, in desert ecosystems, water is the most important environmental factor affecting plant distribution and growth. LWC can reflect the water status of plant tissues (Li et al., 2013) and the resistance and adaptation of plants to drought stress (Joly et al., 2017). With the increase of drought degree, soil water content and LWC of plants decreased greatly (Maréchaux et al., 2015; Zhou et al., 2021). Furthermore, LFW can reflect the dehydration resistance of plants. The higher the LWC, the higher the LFW, and the stronger the drought resistance (Zou et al., 2019). Therefore, through the construction of hMDS and wTDS, the representative functional traits can be screened out. The results of this study showed that desert plants adapt to the arid environment mainly by the changes of morphological and physiological traits. Furthermore, by comparing the components in hMDS with those in the wMDS, it was found that woody plants invest more on stem.

### 4.2 Prediction of spatial distribution characteristics of ecosystem functions by functional trait MDS

Spatial heterogeneity is jointly affected by random and structural factors. In geostatistics, a high nugget-sill ratio (*C*_0_ */(C*_0_ *+ C)* > 50%) indicates a high degree of spatial heterogeneity caused by the random factors. If the ratio is close to 100%, it means that the object variables have constant variation, i.e., the spatial heterogeneity comes from random factors (Boerner et al., 2015; Shi et al., 2020). According to the results of this study, the nugget-sill ratios of the raw and predicted values of C, N, and P cycling for the three plots were less than 25%, the functions in each plot had strong spatial autocorrelation, and the spatial variation was mainly influenced by structural factors. This indicates that natural factors (structural factors) such as topography, parent material, and vegetation play important roles in the spatial variation. The study area is in a national nature reserve, where human interference is minimal. Within each plot, soil type, relief, light radiation, temperature, and other conditions are nearly uniform, so structural factors may come from vegetation type and functional traits of plants. Furthermore, a small proportion of random factors may be caused by experimental errors such as sampling.

Plant functional traits are biological regulators of C, N, and P cycling (Wright et al., 2004; Xu et al., 2017; Butler et al., 2018). They can regulate hydrothermal and material redistribution (Campos et al., 2016; Wasternack, 2017) to influence the extent and intensity of C, N, and P cycling in the ecosystem (McCormack et al., 2015). The OK model results showed that the relative contributions of woody and herbaceous plants to the ecosystem functioning C cycle were mainly determined by the plant biomass. In Plot B, the biomass of herbaceous plants dominated for more C stock, while in Plot A, woody plants were the dominant life forms, and in Plot C, woody plants were more deeply rooted and drought tolerant than herbaceous plants, so woody plants participated in carbon cycles with higher carbon stocks in Plots A and C. The contribution of both woody and herbaceous plants to the ecosystem functional N cycle decreased with gradients, potentially due to moisture limitation (Chen X. et al., 2021). Edmondson et al. (2013) concluded that nitrogen accumulation and transport were influenced by soil moisture, and in natural communities with no anthropogenic nitrogen addition, nitrification and denitrification of plant residues would be more intense in wet areas than in arid areas, with wetter areas receiving more nitrogen accumulation (Liu et al., 2020; Zhao et al., 2021). The contribution of woody plants to the ecosystem functional C cycle and the contribution of herbaceous plants to the ecosystem functional C cycle showed overall contrary results in areas ranging from riparian forests to desert margins. Some studies suggested that in grassland ecosystems, phosphorus was slowing down grassland degradation. When herbaceous plants are moisture-limited, phosphorus stocks are also reduced (Liu et al., 2018). Woody, being perennial, retains more nutrients and reduces the phosphorus metabolic loss in extreme conditions (Zhang et al., 2012). At the small scale of desert ecosystems, ecosystem cycles were largely influenced by vegetation type and may be disturbed by animals and microorganisms in the soil (Zhao et al., 2018). Whereas at large scales (a few square kilometers or hundreds of square kilometers), it has been suggested that ecosystem functioning was affected by climate, topography or anthropogenic emissions (Marklein and Houlton, 2012).

The RK model results show that the wMDS and hMDS can improve the prediction accuracy of the spatial distribution of C, N and P cycling. It has been argued that plant community characteristics influence soil C, N, and P cycling and control the decomposition process in ecosystems. Furthermore, the abundance and composition of species or functional groups within a community influence the input and output of soil C (Oelmann et al., 2011; Zhang, 2020; Shen et al., 2021). According to the mass ratio hypothesis, ecosystem function is primarily determined by the traits of the biomass-dominant species in the community (Smith et al., 2001; Wright et al., 2003; Prentice et al., 2014). Therefore, the relative abundance and biomass of plants and their traits may be the main determinants of C, N, and P cycling in the ecosystem. It has also been shown that plant functional traits can influence C cycling in wetland ecosystems (Wang et al., 2010), plant trait combinations influence the diversity of soil decomposers through the diversity of habitat conditions they create. In turn, the diversity of decomposers may significantly affects soil C cycling (Duan et al., 2021). Li and Wang (2021) argued that C and N cycling were related to plant morphological traits, and ecosystem function was strongly influenced not only by dominant species or functional group traits, but also by dominant functional traits. Meng et al. (2017) showed that C cycling was mainly influenced by vegetation type, which could explain 66.10% of the total variation. Duan et al. (2018) showed that the spatial distribution of C cycling was progressively enhanced by vegetation structure as farmlands were returned to forestland. Gong et al. (2017) also reported a significant positive correlation between plant leaves and soil organic matter content in their study on the C cycling. Therefore, it is reasonable that plant functional traits can predict the function of C cycling in ecosystems. Plant functional traits are the structural factors that have the strongest impact on ecosystem functions.

The results of the RK analysis also indicated that the N and P cycles were also predicted more accurately. Phenotypic traits can reflect changes in ecosystem functions, as well as changes in the spatial distribution of ecosystem processes and functions (McIntyre et al., 2009), such as SLW and SLA (Cornelissen et al., 2003). C, N, and P cycling in ecosystems are often affected by traits of multiple plant organs. For example, the C, N, and P concentrations of plant leaves have a stable positive relationship (Rawat et al., 2020). Furthermore, the maximum diameter of stems (Jucker et al., 2016) and the maximum plant height also have a stable positive relationship with the C, N, and P concentrations of plant leaves (Falster et al., 2011). The accumulation and transformation of N and P between plants and soil is a complex process, which is affected by many environmental factors. Under the same climate and habitat conditions, the dynamic changes of vegetation factors and soil factors regulate the input and output of soil N and P, and then affect the accumulation of N and P in the soil (Dijkstra et al., 2012). Therefore, the functional traits of plants can directly reflect the N and P cycling in the ecosystem.

### 4.3 Comparison of regression kriging models

By comparing the prediction results of the PLS, RF, and BPNN models based on the Kriging method, it was found that the RF model can significantly improve the prediction accuracy on ecosystem functions based on the MDS, and it can accurately predict the spatial distribution of C, N, and P cycling in the desert ecosystem. This may be a non-linear relationship between RF analysis of multiple source auxiliary variables (MDS) and C, N, and P cycling through a classification algorithm to obtain a globally optimal solution, which can overcome the defect of the local minimum solution of the BPNN method (Tyralis et al., 2019). Song et al. (2017) compared the accuracy of Support Vector Machine (SVR), BPNN, and RF models in predicting SOM, and showed that the RF model had higher coefficient of determination and prediction accuracy. This is consistent with the results of this study. Furthermore, it was found that the RF model based on the wMDS had a better performance in predicting C, N, and P cycling than the RF model based on the hMDS, enabling global and point specific predictions. Zeng et al. (2014) also showed that the RF model could better reflect the “pure information” changes in the samples and obviously improve the prediction accuracy of the model.

The BPNN model with non-transparency of data operation has strong fault tolerance, but traditional BPNN models are also prone to over-fitting and local optimality (Chen S. Y. et al., 2021). The results of this study confirmed that the BPNN model was very unstable. PLS, on the other hand, had a low prediction accuracy. It may be that the algorithm has difficulty explaining the loading of independent latent variables. It is based on a cross product with the response variables, rather than on correlations between independent variables in conventional factor analysis (Lednev et al., 2018; Wang et al., 2022). Consequently, visualisation results from the non-linear RF and BPNN models combined with kriging demonstrate that the MDS of plant functional traits could be used to predict C, N, and P cycling in ecosystems.

## 5 Conclusion

In this study, the MDSs (hMDS and wMDS) of plant functional traits was constructed. The spatial distribution of C, N, and P cycling in the desert ecosystem in the Xinjiang Ebinur Lake Basin were accurately predicted based on the hMDS and wMDS using linear and non-linear models combined with regression kriging and geostatistical analysis. The wMDS included H, SLA, LDW, LWC, DBH, LW, and LT, and the hMDS included H, SLA, LFW, LWC, LL, and LW. The cross-validation performed in this study showed that the MDS can replace TDS in predicting ecosystem functions, and the constructed MDSs could accurately predict the spatial distribution of C, N, and P cycling in the ecosystem. Furthermore, C, N, and P cycles are strongly spatially autocorrelated due to structural factors, and C, N and P cycles in desert ecosystems do not behave uniformly between different life forms of plants, subject to water limitation. The RK predicted result was highly consistent with the distribution of the raw values.

## Data availability statement

The original contributions presented in the study are included in the article/**Supplementary Material**. Further inquiries can be directed to the corresponding author.

## Author contributions

Conceptualization, YC and JW. Methodology, YC and JW. Software, YC and JW. Validation, YC and JW. Formal Analysis, GL. Investigation and Project Administration, LJ, HL, HW. Resources. All authors contributed to the article and approved the submitted version.
