# Supplementary material for: Prediction of spatial distribution characteristics of ecosystem functions based on a minimum data set of functional traits of desert plants
Source: Front Plant Sci. 2023 Jun 2;14:1131778. doi: 10.3389/fpls.2023.1131778 (PMC10272538; doi:10.3389/fpls.2023.1131778)
Supplement: Supplementary file 1 [file Table_1.docx]

## Supplementary material

Table. S1 Physical and chemical experiments of soil and plants.

| Classification | | Fluency indices | Experimental method |
| --- | --- | --- | --- |
| Soil | C | Soil organic carbon | Potassium dichromate dilution method |
|  | N | Soil total nitrogen  Soil Nitrate Nitrogen  Soil ammonium nitrogen | Nesslerization  Phenol disulfonic acid method  Extraction - indophenol blue colorimetric method |
|  | P | Soil total phosphorus  Soil available phosphorus | Molybdenum antimony resistance colorimetry  Anti-colorimetric method for extraction of molybdenum and antimony |
| Plant | C | Plant organic carbon | Potassium dichromate dilution method |
|  | N | Plant total nitrogen | Nesslerization |
|  | P | Plant total phosphorus | Molybdenum antimony resistance colorimetry |

Table. S2 Plots (A-C) species and frequencies of plant.

| Plots | Species | Frequentness | Species | Frequentness |
| --- | --- | --- | --- | --- |
| A | *Phragmites australis* | 91.71% | *Suaeda microphylla* | 0.19% |
|  | *Apocynum venetum* | 2.9% | *Alhagi sparsifolia* | 0.12% |
|  | *Halimodendron halodendro* | 1.76% | *Reaumuria soongarica* | 0.11% |
|  | *Nitraria tangutorum* | 1.71% | *Haloxylon ammodendron* | 0.04% |
|  | *Achnatherum splendens* | 0.72% | *Salsola collina* | 0.04% |
|  | *Lycium ruthenicum* | 0.42% | *Sonchus oleraceus* | 0.03% |
|  | *Populus euphratica* | 0.23% | *Glycyrrhiza uralensis* | 0.02% |
| B | *Suaeda glauca* | 38.56% | *Nitraria tangutorum* | 1.39% |
|  | *Halocnemum strobilaceum* | 15.03% | *Salsola collina* | 0.42% |
|  | *Alhagi sparsifolia* | 12.2% | *Populus euphratica* | 0.41% |
|  | *Phragmites australis* | 10.4% | *Suaeda microphylla* | 0.18% |
|  | *Aeluropus pungens* | 6.82% | *Ser. Seriphidium* | 0.18% |
|  | *Reaumuria soongarica* | 6.39% | *Kalidium caspicum* | 0.06% |
|  | *Tamarix chinensis* | 3.57% | *Haloxylon ammodendron* | 0.03% |
|  | *Apocynum venetum* | 2.44% | *Chenopodium glaucum* | 0.03% |
|  | *Karelinia caspia* | 1.91% |  |  |
| C | *Suaeda glauca* | 78.6% | *Calligonum mongolicum* | 0.7% |
|  | *Ser. Seriphidium* | 12.47% | *Salsola collina* | 0.7% |
|  | *Alhagi sparsifolia* | 3.86% | *Reaumuria soongarica* | 0.66% |
|  | *Haloxylon ammodendron* | 1.48% | *Phragmites australis* | 0.04% |
|  | *Nitraria tangutorum* | 1.48% |  |  |
